# Newly-formed emotional memories guide selective attention processes: Evidence from event-related potentials

**DOI:** 10.1038/srep28091

**Published:** 2016-06-20

**Authors:** Harald T. Schupp, Ursula Kirmse, Ralf Schmälzle, Tobias Flaisch, Britta Renner

**Affiliations:** 1 Department of Psychology, University of Konstanz, 78457 Konstanz, Germany

## Abstract

Emotional cues can guide selective attention processes. However, emotional stimuli can both activate long-term memory representations reflecting general world knowledge and engage newly formed memory representations representing specific knowledge from the immediate past. Here, the self-completion feature of associative memory was utilized to assess the regulation of attention processes by newly-formed emotional memory. First, new memory representations were formed by presenting pictures depicting a person either in an erotic pose or as a portrait. Afterwards, to activate newly-built memory traces, edited pictures were presented showing only the head region of the person. ERP recordings revealed the emotional regulation of attention by newly-formed memories. Specifically, edited pictures from the erotic compared to the portrait category elicited an early posterior negativity and late positive potential, similar to the findings observed for the original pictures. A control condition showed that the effect was dependent on newly-formed memory traces. Given the large number of new memories formed each day, they presumably make an important contribution to the regulation of attention in everyday life.

Across their lifespan, humans accumulate emotional memories from a vast number of events and their associated consequences for the self and others, approving or disapproving actions of agents, or the liking or disliking of objects^1^. The foundation of emotional memory is presumed to lie in an associative network that connects sensory-perceptual, conceptual-meaning, and response-action nodes^2^,^3^. These networks are recruited whenever an incoming stimulus matches a stored memory representation. The activation of these emotional networks provides an important signal to direct selective attention processes. Being able to rapidly extract critical information from incoming stimuli facilitates the efficient preparation and organization of appropriate behavioral responses^4^,^5^,^6^.

One kind of emotion network reflects general world knowledge based on stable representations in long-term memory^7^. Presenting prototypical emotional stimuli, i.e., natural scenes and facial expressions, seems to elicit emotional responses in this way^8^,^9^,^10^. Specifically, emotionally diagnostic sensory-perceptual features may ignite a matching memory representation, which in turn commands attentional resources due to the connectivity of the network^2^,^3^. This notion is supported by a large array of neuroimaging studies^9^,^10^: In particular, event-related brain potential (ERP) studies demonstrate that the processing of high-arousing emotional compared with low-arousing control images is associated with an early posterior negativity (EPN), i.e., a negative-going potential over temporo-occipital sensor regions, around 150–300 ms poststimulus, and an enlarged late positive potential (LPP) between 300 and 700 ms poststimulus^9^,^11^,^12^,^13^,^14^,^15^,^16^,^17^,^18^,^19^,^20^. Thus, similar to explicit attention effects^17^, intrinsic stimulus significance modulates ERPs with distinct polarity, topography, and timing across processing stages.

However, while prototypical emotional stimuli representing general world knowledge may be formed slowly and over extended periods of time, emotional networks can also be formed quickly. Milner^21^ noted that humans almost instantaneously build new associations on a daily basis, for instance when we meet new people^21^. Rapid memory formation is critical for distinguishing the general category from specific exemplars, i.e., beautiful people and my beautiful neighbor. In a related way, Conway^22^ suggested that humans form hundreds of new episodic memories each day, binding sensory-perceptual, conceptual, and affective elements^22^. Keeping a sensory-near record of the immediate past facilitates efficient behavior organization by representing context- and situation specific information. In contrast to emotional networks reflecting general world knowledge, little is known with regard to the emotional guidance of attention by newly-formed memories.

The main challenge in studying emotional attention effects by newly-formed memory regards the characteristics of the stimulus materials. Prototypical emotional stimuli cannot disambiguate whether an attention effect stems from a new or an old memory representation. The self-completion feature of associative networks makes it possible to activate emotional memories while excluding potential confounds due to preexisting emotional memory representations. Specifically, activating only some members of the ensemble, i.e., by partial input, can ignite the entire network^23^ via recurrent processing and self-completion. Thus, when emotional networks are newly-formed, the presentation of partial input devoid of emotionally diagnostic information can be used to activate newly-formed memory representations while preventing the activation of lasting emotional memory representations. The self-completion characteristic of memory networks has been studied in previous research on implicit recognition processes. Forming memory representations by stimulus exposure facilitates recognition of fragments or partially occluded objects in the absence of intention and explicit task instructions^24^,^25^,^26^,^27^. Here we apply this approach to the emotion domain and present partial input to ensure that emotional attention effects reflect newly-formed rather than old memories.

The present study examined the hypothesis that newly-formed emotion networks can guide selective attention processes. To form new memory representations, erotic and portrait pictures were repeatedly presented in a passive viewing task. For this first phase, we predicted larger EPN and LPP amplitudes to erotic images. To be able to isolate newly-formed memories in Phase 2, pictures from both categories were edited to show only the head region of the person (see Fig. 1). Thus, the partial pictures were able to activate newly-formed memory traces from the two stimulus categories. Critically, the primary difference in emotional information between the stimulus categories was occluded, thereby preventing the activation of general emotion knowledge associated with erotica. According to the hypothesis that newly-formed emotion networks can guide attention processes, partial stimuli from the erotic stimulus category were predicted to elicit larger EPN and LPP components as compared to the portrait category. A control condition assured that this effect, predicted for previously ‘seen’ partial images, involved memory processes: Specifically, the EPN and LPP component towards novel partial images, which were not seen during phase 1, should not differ between the erotic and portrait picture categories.

## Results

### Partial pictures: Recognition and explicit categorization data

Participants were highly accurate (99.2%) and confident (M = 6.92) when asked to decide whether a partial picture belonged to a whole picture seen in Phase 1. Similar results emerged when participants categorized the pictures seen as a whole image in Phase 1 for nudity (96.9% correct, confidence M = 6.8). With regard to the partial pictures not seen in Phase 1, a category difference was observed. While judgment of whether the person was nude or dressed was at chance for partial images from the erotic category (51.6% correct), participants achieved a significantly better hit rate (75.8% correct) for images from the portrait category, *t*_31_ = −4.0, *P* < 0.0001, *d* = 0.71. As expected, confidence when categorizing novel partial pictures as nude or dressed was significantly reduced (M = 4.4) as compared to partial pictures seen in Phase 1, *F*_1,31_ = 126.8, *P* < 0.0001, *η*^2^ = 0.81.

### Event-related potentials

#### Phase 1: Whole image processing

Whole image processing replicated previous findings regarding EPN and LPP components. Specifically, while the ERP waveform presented a positive polarity over posterior sensors, the processing of erotic as compared to portrait pictures resulted in a relative negative shift in the ERP waveform over posterior sensor sites, as illustrated in Fig. 2A by the waveforms for a representative occipito-temporal sensor and a difference map (erotica – portrait) representing the mean activity from 172–272 ms. Subsequently, the difference in processing erotic and portrait pictures was captured by a relative positive shift over centro-parietal sensor sites, with Fig. 3A displaying waveforms for a representative central sensor and difference scalp maps representing mean activity form 352–452 ms. Statistical analysis supported the significance of these findings for the EPN, *t*_31_ = −6.8, *P* < 0.0001, *d* = 1.2, as well as the LPP component, *t*_31_ = 4.8, *P* < 0.0001, *d* = 0.84.

#### Phase 2: Partial image processing

EPN and LPP components associated with the processing of partial pictures were pronouncedly modulated by the factor *Stimulus Experience*. For pictures seen in Phase 1, compared to the portrait images, partial pictures from the erotic stimulus category elicited modulations in the EPN and LPP components similar with respect to latency, topography, and polarity as observed in Phase 1, when the whole images were presented (see Figs 2B and 3B). Critically, the EPN and LPP effects were dependent on seeing the whole pictures in Phase 1. No EPN and LPP difference emerged between the stimulus categories when the partial pictures were novel (see Figs 2C and 3C).

##### Early posterior negativity (EPN)

A significant interaction between *Stimulus Experience* and *Picture Category* was observed, *F*_1,31_ = 4.6, *P* < 0.05, *η*^2^ = 0.13. Post-hoc tests revealed a relative negative potential over posterior sensor sites for partial pictures from the erotic as compared to the portrait category, *t*_31_ = −2.8, *P* < 0.01, *d* = 0.49, when the partial pictures had been seen as whole images in Phase 1. In contrast, when the partial pictures were novel, the EPN showed a relative positive rather than negative potential difference, which however, was not significant, *t*_31_ = 1.3, *P* = 0.2.

##### Late positive potential (LPP)

The interaction of *Stimulus Experience* and *Picture Category* was significant, *F*_1,31_ = 4.9, *P* < 0.05, *η*^2^ = 0.14. When the partial pictures had been seen as whole images in Phase 1, a relative positive potential was observed over centro-parietal sensor sites for partial pictures from the erotic as compared to the portrait stimulus categories, *t*_31_ = 3.7, *P* < 0.001, *d* = 0.66. In contrast, when the partial pictures were novel, there was no significant modulation of the LPP component for pictures from the two stimulus categories, *t*_31_ = −0.7, *P* = 0.49.

##### Sustained posterior positivity

While novel partial pictures revealed no hint of an emotional modulation of the EPN and LPP components, an unexpected ERP difference between unseen erotic and portrait partial pictures was observed. Specifically, a sustained positivity was observed between 200 and 500 ms over parieto-occipital sensor sites for partial pictures from the erotic as compared to the portrait stimulus category (see Supplementary Fig. 1), *t*_31_ = 4.1, *P* < 0.001, *d* = 0.71. There was no effect for seen partial pictures, *t*_31_ = 0.7, ns, and the interaction of *Stimulus Experience* and *Picture Category* was significant in the overall analysis, *F*_1,31_ = 4.3, *P* < 0.05, *η*^2^ = 0.12.

#### Phase 1 vs. Phase 2: Emotional modulation during whole and partial picture processing

Inspection of Figs 2 and 3 suggests that the emotional modulation of the EPN and LPP is stronger for pictures presented as whole images as compared to partial pictures previously seen in Phase 1. However, statistical analysis revealed that stronger emotional modulation effects for whole than partial picture processing is statistically sound for the EPN component only. Specifically, the difference between erotic and portrait images was significantly more negative (M = 0.70, SD = 1.15) for the EPN component, *t*_31_ = −3.4, *P* < 0.01, *d* = 0.61, while not reaching significance for the LPP component (M = 0.37, SD = 1.85), *t*_31_ = 1.1, *ns*.

## Discussion

Emotional cues play an important role in the regulation of the attentional spotlight^6^,^8^,^9^,^10^. The present study was designed to examine the emotion-attention relationship with respect to newly-formed memory representations. The findings revealed the prototypical brain signature of attentive processing for new emotional memory representations of information at the level of the individual person. Specifically, the EPN and LPP components were larger for partial images from the erotic as compared to the portrait category. These findings extend the regulation of attention by emotional cues to the vast number of episodic memories formed each day^22^.

**Figure 1:**
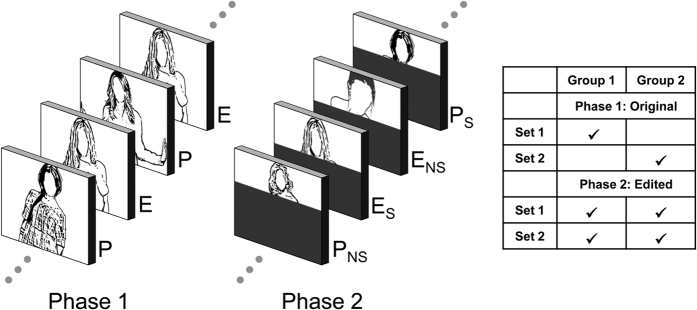
Illustration of the study design. During phase 1, participants viewed pictures from two stimulus categories: Erotica (E) and Portraits (P). During phase 2, participants viewed partial pictures created by occluding each picture below the neck of the person. Depending on whether participants could or could not form a memory representation of the face when viewing the picture in phase 1, four categories were distinguished: Erotica-Seen (E_S), Erotica-Not Seen (E_NS), Portrait-Seen (P_S), and Portrait-Not Seen (P_NS). The right figure illustrates the counterbalancing of the picture sets.

When investigating the role of newly-formed memory in directing attention processes, the potential confound of stable, long-lasting memory representations has to be avoided. Specifically, an attention effect observed for stimuli depicting emotionally diagnostic information may depend on long-lasting general world knowledge rather than newly-formed memory representations. This reasoning is supported by the observation that emotional stimuli novel to the observer elicit a larger EPN and LPP component, as previously shown for natural scenes^14^,^17^,^18^, facial expressions^16^,^19^,^28^,^29^, words^15^ and gestures^11^,^12^. One solution to this problem is provided by the self-completion characteristic of an associative network, i.e., the propensity of a partial cue to activate the entire network^23^. As shown in Fig. 1, the presence of emotionally diagnostic information was occluded in the critical condition of the present study, making partial pictures from the erotic and portrait category similar in appearance. Yet the remaining cues, i.e., the head of the person, were sufficient to activate an individual memory representation for each depicted person. Furthermore, the study design included a control condition examining the processing of novel partial images, which could not activate recently formed memory representations. Specifically, counterbalanced across participants, half the partial pictures presented during phase 2 were ‘not seen’ during phase 1. As expected, the presentation of these partial images showed no emotional differentiation of the EPN and LPP component. Accordingly, any difference in attention devoted to the previously ‘seen’ partial erotic and portrait images can be attributed to the activation of newly formed memory representations.

**Figure 2:**
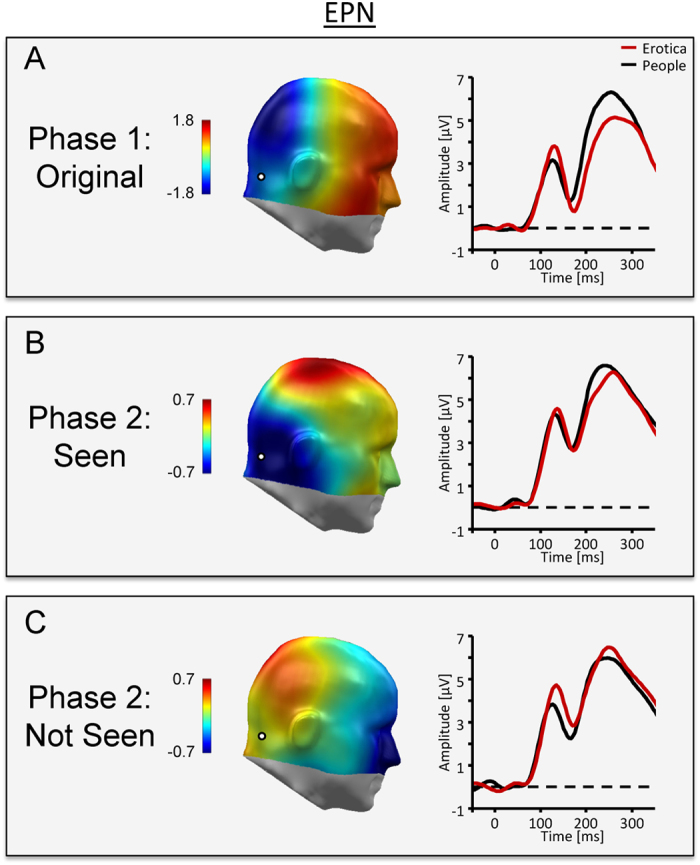
ERP waveforms for a representative occipito-temporal sensor (EGI#167, scalp position marked by white dot) and difference scalp maps [erotic – portrait images] illustrate the EPN component across experimental conditions. (**A**) Phase 1 illustrates the prototypical ERP signature of emotion processing. Erotic images elicited a larger posterior negativity than portrait pictures. (**B**) Consistent with the self-completion hypothesis, edited pictures from the erotic category elicited an emotional modulation of the EPN. (**C**) The effect was dependent on newly-formed memory representations as partial pictures which had not been seen in their original form did not elicit an emotional modulation as indicated by the EPN.

**Figure 3:**
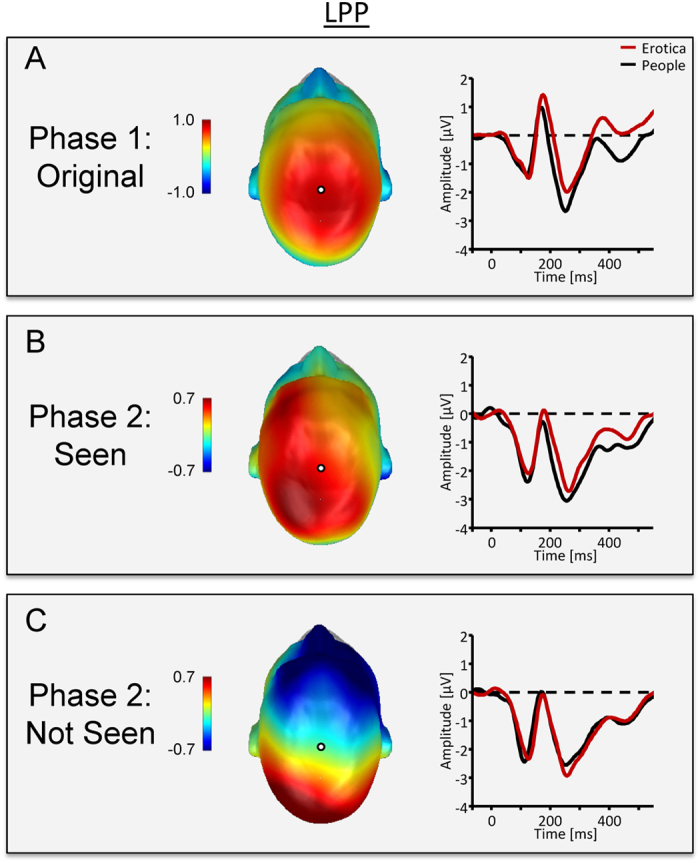
ERP waveform for a representative central sensor (EGI #81, scalp position marked by white dot) and difference scalp maps [erotic – portrait images] illustrate the LPP component across experimental conditions. (**A**) In Phase 1, erotic images elicited a larger LPP than portrait pictures. (**B**) Consistent with the self-completion hypothesis, edited pictures from the erotic category elicited a larger LPP than portrait pictures. (**C**) The effect was dependent on newly-formed memory representations as partial pictures which had not been seen in their original form did not elicit an emotional modulation as indicated by the LPP.

The results of phase 2 support the hypothesis that newly-formed emotional memories guide attention processes similarly as stable, long-term memory representations^9^. Partial images from the erotic category elicited larger EPN and LPP components as compared to partial images from the portrait category (see Figs 2B and 3B). Note that the EPN and LPP components elicited by partial pictures were analyzed using the sensor clusters and time windows derived from processing the whole pictures in phase 1. Thus, by assuring that the associated ERP components corresponded in defining features, i.e., in latency, topography, and polarity, this approach strengthens the finding that newly-formed memories can drive attention processes. The main difference in emotional ERP modulations between whole and partial picture processing regarded the strength of the effects. For the EPN component, the effect was stronger for whole pictures, presumably indicating a stronger match with memory representations when pictures show emotionally diagnostic information, i.e., sexual features^3^. The finding that the difference between whole and partial pictures mainly affected the early processing period at which motivationally significant stimuli are presumably “tagged” for preferential processing awaits replication. If confirmed, it would add further evidence that the two ERP components, i.e., EPN and LPP, modulated by emotional significance distinguishably respond to antecedent stimulus and task conditions^9^. Of note, there appears to be little difference in the onset latency of the emotional modulation of the EPN during whole and partial image processing. This finding presumably reflects remarkably efficient processes of stimulus identification and categorization, indicated by research on natural scene processing under challenging conditions, i.e., short exposure times, and dual task conditions^30^,^31^. Furthermore, ERP differences between target and non-target stimuli emerge around 150 ms, indicating an upper limit for the time needed to distinguish between higher-order semantic stimulus categories^31^,^32^. Accordingly, it appears that the self-completion of newly-formed memory representations, activated by partial input, occurs rather fast, resulting in no latency delay in emotional modulation effects as compared to the presentation of whole pictures displaying emotionally diagnostic information. Overall, the self-completion characteristic of associative networks provides a promising avenue to explore newly-formed emotional memories. Given the estimate of hundreds of new memories formed each day^22^, these presumably make an important contribution to the regulation of attention in everyday life.

The present study was derived from an associative network perspective in which emotion networks are characterized by interlinked sensory-perceptual, conceptual-meaning, and response-action nodes. The network is activated when stimuli match a memory representation eliciting the spectrum of measurable physiological, verbal, and behavioral emotion responses^2^,^3^. The novel aspect of the present research is that the formation of specific sensory-perceptual representations, i.e., at the level of the individual person, is able to ‘ignite’ a network comprising emotional response nodes. Specifically, it is presumed that the repeated exposure to the stimulus materials leads to the formation of new associations in perceptual representation systems^26^. According to previous research and theory, this may reflect an implicit memory which is formed in the absence of intention and explicit task instructions^26^. It has been well established that stimulus exposure facilitates perceptual recognition processes underlying the ability to recognize fragments or partially occluded objects^25^,^27^,^33^. The present study extends these measures of implicit memory in providing first evidence that newly-formed memory representations of individual persons can elicit a characteristic response associated with emotional stimuli, i.e., the guidance of attention processes.

Although speculative, it may be informative to relate the present findings to the broader conception of episodic memory^21^,^22^,^34^. For instance, Conway conceptualizes episodic memories as ‘sensory-perceptual-affective-conceptual short-time slice records of experience’^34^^, p. 219^ which contextualize general (semantic) world knowledge in order to reach short-term goals^22^. Viewed from this perspective, general knowledge about people, events, and situations stored in long-term memory can be complemented by knowledge about the specific instance. The rapid formation of new representations when making new acquaintances and the updating of socially-relevant information about the plans and moods of family, friends, and others seems to be important for social interaction. Interestingly, there is some evidence that episodic memories become inaccessible rather than lost and that providing sensory-perceptual cues in the form of photographs can restore the accessibility of episodic memories^22^. Thus, future studies need to detail the temporal relationship between the sensory-perceptual memory representations in the posterior brain and the anterior cortical and subcortical brain regions presumably involved in representing affective-conceptual meaning as well as the relationship to episodic memory.

The ignition of an emotion network by partial input has been mainly discussed in the context of psychopathology. Victims of post-traumatic stress disorder report that seemingly trivial sensory impressions can trigger flashback experiences of the trauma, including similar vegetative and behavioral responses^35^. It has been suggested that trauma memory consists of low-level stimulus representations, including sensory-perceptual-affective stimulus qualities, lacking the (usual) association with autobiographical and contextual information^36^,^37^. The strength of the emotion involved, the presence of fragmented and disorganized memory, and the perceived distress by the patients may have obscured the recognition of a basic operation principle of emotional networks: Experience rapidly forms new memories in which sensory-perceptual-affective-meaning units represent the immediate past and regulate attention processes. Noteworthily, memory representations were established in the present study by merely asking participants to view the pictures. Thus, there were no traumatic events involved, as in the case of flashback memories in PTSD patients, nor were strong conditions for learning realized as is the case in associative, observational and model learning^38^,^39^,^40^.

An unexpected finding was that the processing of novel erotic as compared to portrait partial pictures was associated with a distinct ERP effect, i.e., a positivity over parieto-occipital sensors between 200 and 500 ms. The intermixed presentation of known and novel partial images may have invited guessing and a superior performance was observed in assigning novel partial images to the portrait as compared to the erotic stimulus category, presumably due to residual information. In all likelihood, this effect thus relates to aspects of stimulus classification and recognition memory^41^,^42^. Modifying the study design, i.e., comparing pre-post processing of partial images and forming new memories in between the two phases, can address the issue. Of most relevance, the parietal positive component was characterized by a distinct morphology, i.e., in latency and topography, as compared to the emotion-modulated EPN and LPP components, thereby supporting the main conclusion of the study.

There are some limitations to this study. First, the present study focused on male participants and visual stimuli associated with sexual reproduction. The rationale for this approach was to increase the sensitivity of the experimental paradigm by studying stimulus materials proven in previous research to consistently and reliably elicit responses in somatic, autonomic, and neuroimaging measures^9^,^43^,^44^, and these are most accentuated in male participants^45^,^46^. Future studies need to corroborate the present findings studying both genders and a broader range of pleasant and unpleasant stimulus materials. Furthermore, to examine the notion that new memories can be formed instantaneously^21^,^22^, future studies need to systematically vary the number of stimulus repetitions. Finally, given the lasting effects of priming^24^,^26^, studies assessing the sustained effects of newly-formed emotion memories appear highly informative.

## Conclusion

Researchers often distinguish between multiple memory systems based on distinct functions, neural correlates and processing characteristics. Previous research primarily examined the regulation of attention by emotional cues based on general world knowledge stored in long-term memory. The present research extended the traditional approach to the domain of newly-formed emotional memory representations relying on the self-completion characteristic of associative networks to disentangle newly-formed from stable emotional representations. The results support the notion that newly-formed individual memory representations guide selective attention processes as indexed by the EPN and LPP components. Regulating attention focus according to newly-formed emotional memories seems to be an important mechanism in everyday life.

## Materials and Methods

### Participants

Thirty-two male volunteers aged between 18 and 31 years (M = 22.4, SD = 3.1) participated in the study. Sample size had been determined in advance and data collection was stopped when the planned sample size was reached. Participants received monetary compensation or course credit for participation. The ethical committee of the University of Konstanz approved the experimental procedure in accordance with the regulations of the Declaration of Helsinki, and all methods were carried out in full compliance with the approved guidelines. All participants provided informed consent.

### Stimuli

#### Whole images

The ‘Whole’, or unedited, stimulus materials comprised two picture categories (N = 16). Both picture categories depicted a single female person in the center of the picture. The critical difference between the categories regarded sexual explicitness. The erotic stimulus category consisted of images showing a single female with visible sexual features, similar to erotic art and photography (N = 8). The portrait category showed a single female person dressed and in a pose similar to portrait photographs (N = 8).

#### Partial images

The pictures comprising the ‘Whole’ image set were edited to create the ‘Partial’ images. As shown in Fig. 1, each image from the ‘Whole’ image set was overlaid with a grey box to occlude all visual information below the neck. As a result, only the face of the female person and surrounding background information was retained and no explicit sexual features were visible.

### Procedure

As shown in Fig. 1, the experiment consisted of two phases. In Phase 1, participants viewed pictures from the ‘Whole’ image set. To manipulate the factor *Stimulus Experience*, the stimulus set was divided in two subsets, with each participant viewing one subset in Phase 1, counterbalanced across participants. Pictures (N = 8) were repeated twenty-five times, resulting in a total of 200 presentations. Previous studies revealed little evidence for the habituation of emotion processing in terms of EPN and LPP modulation^20^,^47^. Thus, in order to enhance familiarity and experience with the stimulus materials, a small number of stimuli can be used while maintaining sensitivity to demonstrate selective emotion processing.

In Phase 2, each participant viewed pictures from both partial image sets. Thus, for each participant, half of the partial images had already been presented in their original form in Phase 1 (‘Seen’), while the other half had not been seen in the full version (‘Not Seen’). Each picture (N = 16) was presented twenty-five times in random order, resulting in a total of 400 presentations. To prevent the contribution of differences in physical stimulus characteristics, the picture subset seen in Phase 1 was counterbalanced across participants (see Fig. 1). As a result, individual pictures from the two picture sets contributed equally to each of the four conditions of interest (i.e., Erotic-Seen, Erotic-Not Seen, Portrait-Seen, Portrait-Not Seen).

Pictures were displayed in both phases for 1 s followed by an inter-trial interval varying from 1 to 1.9 s (M = 1.45 s). In both phases, the order of picture presentation was pseudo-random with no more than three repetitions of stimuli from the same category allowed. Each participant viewed a different order of picture presentation with transition frequencies between all categories controlled^13^. A short break (~5–10 minutes) allowed posture adjustment during the two phases, which lasted approximately 8.5 and 17 minutes, respectively. Participants were instructed to simply view the pictures. Following the second phase, participants provided several ratings and were debriefed.

### Self report measures

Participants provided ratings for the partial pictures presented in Phase 2 to assess picture recognition and explicit category knowledge. Specifically, they made Yes/No judgments as to whether (1) the picture had been seen in Phase 1 and (2) depicted a nude or dressed person, and (3) provided confidence ratings for these two judgments on a scale ranging from 1 to 7.

After rating the partial images, participants evaluated all pictures from the ‘Whole’ image set according to their perceived pleasantness and arousal using the Self-Assessment Manikin on a scale ranging from 1 to 7^48^. A 2 (erotic vs. portrait picture category) ×2 (picture set 1 vs. picture set 2) ANOVA indicated that erotic images were perceived as more pleasant (M = 6.5, SD = 1.0) compared to portrait pictures (M = 5.8, SD = 0.7), *F*_1,31_ = 16.0, *P* < 0.0001, *η*^2^ = 0.34. Furthermore, erotic images (M = 6.2, SD = 1.5) were evaluated as more arousing compared to the portrait picture category (M = 3.7, SD = 1.3), *F*_1,31_ = 103.1, *P* < 0.0001, *η*^2^ = 0.77. There were no main effects or interactions involving the factor *Picture Set*.

### ERP data acquisition

Brain and ocular scalp potential were measured with a 256 lead geodesic sensor net (HCGSN), on-line bandpass filtered from 0.1–100 Hz, and sampled at 250 Hz using Netstation acquisition software and EGI amplifiers (Electrical Geodesics, Inc. Eugene, OR). Electrode impedance was kept below 40 kΩ, as recommended for this type of electroencephalogram (EEG) amplifier by EGI guidelines. Data were recorded continuously with the vertex sensor as reference electrode. A 40 Hz digital low pass filter was applied off-line to the continuous EEG data. The reported data were corrected for ocular artifacts based on a multiple regression method^49^, converted to an average reference, and baseline-adjusted (100 ms pre-stimulus). Data editing and artifact rejection were performed based on an elaborate method for statistical control of artifacts, specifically tailored for the analyses of dense sensor ERP recordings^50^. Finally, separate average waveforms were calculated for the two experimental conditions in Phase 1 (*Picture Category*: Erotic vs. Portrait) and the four experimental conditions in Phase 2 (*Picture Category*: Erotic vs. Portrait; *Stimulus Experience*: Seen vs. Not Seen). Applying strict artifact criteria, on average 78.1% of the trials were used to calculate the average waveforms, which did not differ across experimental conditions (SD = 0.9).

### ERP analysis

#### Phase 1: Whole image processing

Visual inspection of the waveforms revealed the replication of the basic finding that erotic as compared to portrait pictures elicited EPN and LPP effects with the predicted latency, topography, and polarity. To capture the EPN component over left and right hemispheric sites, mean amplitudes from representative temporo-occipital sensors were averaged across a time interval from 172–272 ms (EGI sensor numbers of the left cluster: 74, 75, 76, 83, 84, 85, 92, 93, 94, 95, 96, 97, 102, 103, 104, 105, 106, 107, 111, 112, 113, 114, 120, 121, 122, 133, 134; right cluster: 160, 161, 166, 167, 168, 169, 170, 171, 172, 174, 175, 176. 177, 178, 179, 180, 187, 188, 189, 190, 191, 192, 199, 200, 201, 208, 209). Please note that according to previous research the overall ERP waveform can appear markedly different depending on stimulus materials (natural scenes, faces, gestures, and words) and experimental paradigm (e.g., rapid and continuous picture presentation vs. slow presentation and long ITI)^9^,^11^,^12^,^13^,^14^,^15^,^16^,^17^,^18^,^19^,^20^. Nonetheless, these various emotional stimuli are uniformly associated with an EPN, i.e., a negative ERP difference relative to control stimuli over posterior sensor regions^17^. Furthermore, with the notion of a stronger positive potential for neutral compared to emotional materials, some readers might infer stronger neural activation for neutral materials. However, fast picture presentation fMRI-studies revealed increased activation of the extended visual cortex by emotional pictures poststimulus^51^.

To assess the LPP component over left and right hemispheric sites, the mean activity from representative centro-parietal sensors were averaged across a time interval from 352–452 ms (EGI sensor numbers of the left cluster: 9, 17, 24, 42, 43, 44, 45, 50, 51, 52, 53, 58, 59, 60, 65, 66, 72, 77, 78, 79, 80; right cluster: 131, 132, 143, 144, 154, 155, 163, 164, 173, 182, 183, 184, 185, 186, 195, 196, 197, 198, 205, 206, 207).

EPN and LPP components data were submitted separately to repeated measures analysis of variance (ANOVA), including the within subject factors of *Picture Category* (Erotic vs. Portrait) and *Laterality* (Left and Right Sensor Clusters).

#### Phase 2: Partial image processing

The main hypothesis of the study was that partial images from the erotic and portrait stimulus category, which had been seen as whole images in Phase 1, elicit a similar ERP modulation as observed in Phase 1 for the whole images. Accordingly, the ERP scoring of partial image processing in Phase 2 was conducted with reliance on the same sensor cluster and time windows used to determine EPN and LPP components during whole picture processing in Phase 1. Supplementary information regarding L2- minimum norm estimate and current source density analyses are provided in the Supplementary Fig. S2. Furthermore, visual inspection revealed an unpredicted category effect specifically observed for novel pictures. The effect appeared as sustained parieto-occipital positivity and was scored as mean activity over a time interval from 200–500 ms in left and right temporo-occipital sensor clusters (EGI sensor numbers of the left cluster: 106, 107, 108, 115, 116, 117, 118, 124, 125; right cluster: 127, 138, 139, 149, 150, 151, 159, 160, 169,).

Initial analysis of the data revealed no effects of interest with regard to the factor *Laterality*. Thus, for brevity, the factor of *Laterality* was dropped from further consideration, and reported findings are based on the average of left and right sensor clusters of the EPN and LPP components. Both ERP components were separately submitted to repeated-measures ANOVA analysis, including the within subject factors *Picture Category* (Erotic vs. Portrait) and *Stimulus Experience* (Seen vs. Not Seen). Significant interactions of *Stimulus Experience* x *Picture Category* were followed up by separate t-tests for the Seen and the Not Seen condition, respectively, comparing the EPN and LPP amplitudes for erotic versus portrait stimuli.

#### Phase 1 vs. Phase 2: Emotional modulation during whole and partial picture processing

Additional analyses compared the emotional modulation of the EPN and LPP for pictures presented as whole images as compared to partial pictures previously seen in Phase 1 by submitting the EPN and LPP difference (erotic – portrait) for these two conditions to a t-test.

When appropriate, the Greenhouse-Geisser procedure was used to correct for violations of sphericity and Bonferroni correction was applied for post hoc tests to control for Type 1 error.

## Additional Information

**How to cite this article**: Schupp, H. T. *et al*. Newly-formed emotional memories guide selective attention processes: Evidence from event-related potentials. *Sci. Rep.* **6**, 28091; doi: 10.1038/srep28091 (2016).

## Supplementary Material

Supplementary Information
