# Supplementary material for: Newly-formed emotional memories guide selective attention processes: Evidence from event-related potentials
Source: Sci Rep. 2016 Jun 20;6:28091. doi: 10.1038/srep28091 (PMC4913271; doi:10.1038/srep28091)
Supplement: Supplementary Information [file srep28091-s1.doc]

**Supplementary Information**

**Newly-formed emotional memories guide selective attention processes: Evidence from event-related potentials**

**Harald T. Schupp, Ursula Kirmse, Ralf Schmälzle, Tobias Flaisch & Britta Renner**

**Department of Psychology, University of Konstanz, 78457 Konstanz, Germany**

Correspondence to: Harald.Schupp@uni-konstanz.de

**Supplementary Figure 1:**

**Sustained posterior positivity**


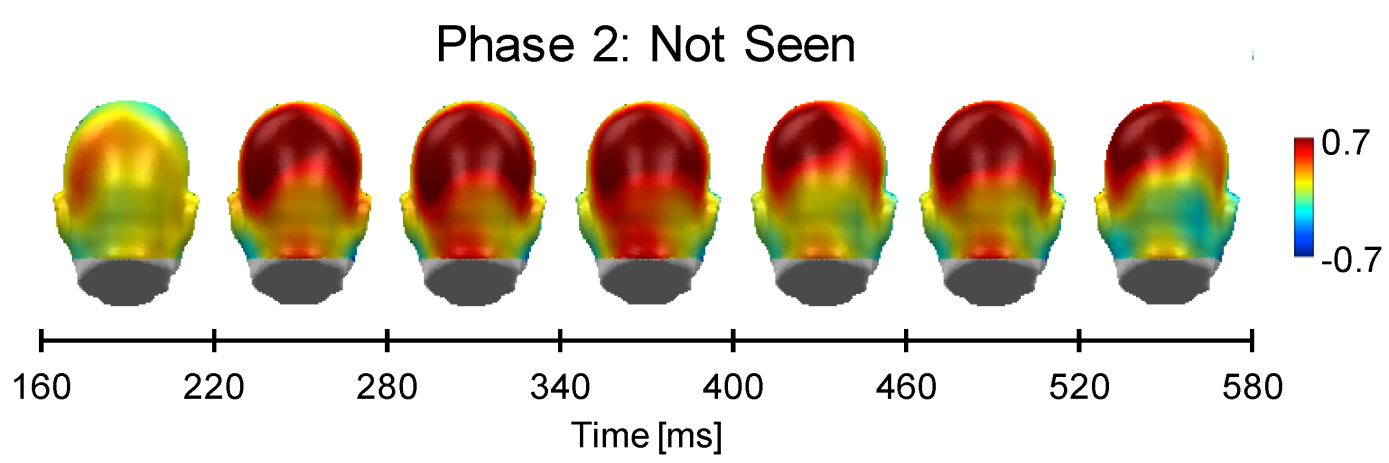


Supplementary Figure 1. Difference scalp maps illustrate the sustained positivity over parieto-occipital sensor sites for partial pictures from the erotic as compared to the portrait category, when the partial pictures had not been seen as whole images in Phase 1.

**Supplementary Figure 2:**

**Minimum Norm Estimate and Current Source Density analysis**


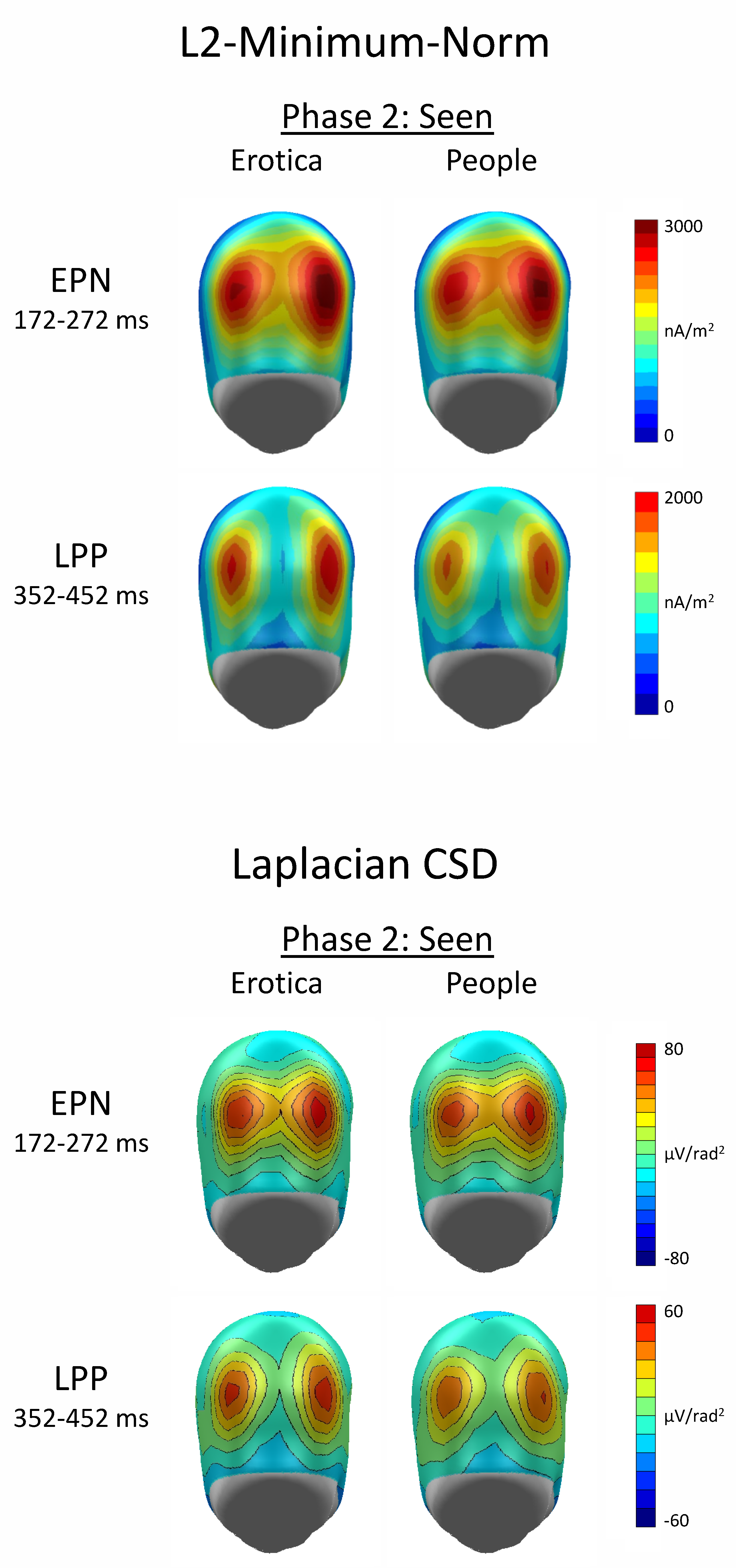


Supplementary Figure 2. Topographical distributions of the (top panel) L2-minimum norm estimate (L2-MNE; source model: 3 (radial, azimutal and polar direction) x 197 dipoles, shell radius: 8 cm) and (bottom panel) current source density (Laplacian CSD) for partial erotic and portrait images in the EPN (172-272 ms) and the LPP (352-452 ms) time windows, when the partial picture had been seen as whole images in Phase 1. Statistical analysis confirmed significant differences between partial erotic and portrait images for L2-MNE and CSD analyses and both time windows, *t*’s_31_> 2.4, *P*’s < .05.
